# Exploring the Potential of *Torulaspora delbrueckii*, *Starmerella bacillaris*, and *Saccharomyces cerevisiae* as a Probiotic Starter for Craft Beer Production

**DOI:** 10.3390/foods14091608

**Published:** 2025-05-01

**Authors:** Carmen-Rodica Vrînceanu, Filofteia Camelia Diguță, Mihaela Dragoi Cudalbeanu, Alina Ortan, Constanța Mihai, Iuliana Diana Bărbulescu, Mihai Frîncu, Mihaela Begea, Florentina Matei, Răzvan Ionuț Teodorescu

**Affiliations:** 1 Faculty of Land Reclamation and Environmental Engineering, University of Agronomic Sciences and Veterinary Medicine of Bucharest, 59, Mărăști Blvd., District 1, 011464 Bucharest, Romania; carmen_stoica@yahoo.com (C.-R.V.); mcudalbeanu@gmail.com (M.D.C.); alina.ortan@fifim.ro (A.O.); constanta.mihai@fifim.ro (C.M.); razvan.teodorescu@usamv.ro (R.I.T.); 2 Faculty of Biotechnologies, University of Agronomic Sciences and Veterinary Medicine Bucharest, 59, Mărăști Blvd., District 1, 011464 Bucharest, Romania; camelia.diguta@bth.usamv.ro (F.C.D.); diana.barbulescu@bth.usamv.ro (I.D.B.); florentina.matei@unitbv.ro (F.M.); 3 Research Center for Studies of Food Quality and Agricultural Products, University of Agronomic Sciences and Veterinary Medicine of Bucharest, 59, Mărăști Blvd., District 1, 011464 Bucharest, Romania; 4 Faculty of Biotechnical Systems Engineering, National University of Science and Technology Politehnica Bucharest, 313 Splaiul Independenței, 060042 Bucharest, Romania; 5 Faculty of Food Industry and Tourism, Transilvania University of Brașov, 148 Castelului St., 500014 Brașov, Romania

**Keywords:** *Saccharomyces cerevisiae*, non-*Saccharomyces*, screening, probiotic traits, brewing potential, micro-fermentations, craft beer

## Abstract

This paper explores the broad probiotic and functional properties of two non-*Saccharomyces* strains (MI120 and MI125) and one *Saccharomyces cerevisiae* BB06 strain (as a reference probiotic). *Torulaspora delbrueckii* MI120 and *Starmerella bacillaris* MI125 were identified via 5.8S rDNA sequencing. All the strains survived well in simulated gastrointestinal conditions and had strong antioxidant activity (>68%). *S. bacillaris* MI125 excelled in antimicrobial activity against Gram-positive and Gram-negative bacteria. *S. bacillaris* MI125 and *Sacch. cerevisiae* BB06 resisted all the tested antibiotics. No strain displayed hemolytic behavior. The freeze-dried yeast strains achieved survival rates between 76.62% and 93.38%. Based on our physiological characterization analysis (carbon assimilation, ethanol tolerance, acetic acid and H_2_S production, temperature and low pH tolerance, enzymatic pattern, and killer phenotype), all the strains showed interesting attributes, with *Sacch. cerevisiae* BB06 fermenting vigorously in malt extract medium. Beer fermented with *T. delbrueckii* MI120 had the highest phenolic content (96.02 μg GAE·mL^−1^) and antioxidant activity (90.43%), matching commercial *Sacch. cerevisiae* US-05 in sensory traits such as taste and aroma. However, after two months in bottled beer, the yeast viability decreased to 2–3 log CFU·mL⁻^1^. The pilot brewing and the assessments of the strains’ technological, physico-chemical, and sensorial properties confirmed their suitability for industrial brewing. Overall, *T. delbrueckii* MI120 emerges as a promising brewing strain, and *S. bacillaris* MI125 is a potential probiotic.

## 1. Introduction

The global beer industry produced around 1.88 billion hectoliters in 2023, with China, the United States, and Brazil topping the list of leading producers [1,2]. Beer production within the European Union reached 346 million hectoliters in 2023, with Romania ranking in 10th position with 14.94 million hectoliters [1,2]. Romania’s ranking in 2023 was fifth in EU consumption, with an average consumption of 83 liters of beer per capita [3]. Meanwhile, the EU reported the presence of 3275 active microbreweries in 2023, 85 of which were located in Romania [3,4]. A microbrewery is a small, independent, and traditional brewing establishment, even in regions without a deep-rooted brewing tradition [5,6,7,8,9,10]. The yeast strain and substrate composition primarily determine the flavor and aroma of beer [9,10,11]. The *Saccharomyces* genus plays a crucial role in the fermentation process, where its unique characteristics, combined with the brewers’ expertise, shape the final product [12,13,14,15]. *Sacch. cerevisiae* (ale beer) and *Sacch. pastorianus* (lager beer) are the two main types renowned for their fermentation potential of a variety of sugars, including glucose, fructose, mannose, and maltose, which generates various metabolites that influence the beer’s flavor and the strains’ ability to survive under multiple stress conditions, such as low pH and high ethanol levels [12,13,14]. Craft brewers excel at experimenting with innovative combinations of raw and malted grains while enhancing beer flavors by adding fruits, spices, and other ingredients [6,7,9,10,16,17,18]. These practices result in a distinctive and authentic craft beer [7,9,10]. *Saccharomyces* and non-*Saccharomyces* yeasts, often isolated from wild sources such as vineyards, winemaking environments, sourdough cultures, and Kombucha, contribute valuable biological diversity that enhances the beer’s flavor complexity and compositional profile [13,19,20,21,22,23,24,25,26,27,28,29]. Today, modern trends in brewing have embraced the use of non-*Saccharomyces*, whether individually or in combination with *Saccharomyces* spp. [26,27,28,29,30,31]. Research has demonstrated their ability to influence ethanol, glycerol, higher alcohols, acetaldehyde, and diacetyl levels, thereby facilitating the production of low-alcohol or alcohol-free beers with a distinct aromatic profile [30,31,32,33]. *Pichia kluyveri* has been patented for producing low-alcohol or alcohol-free beer [34]. *Torulaspora delbrueckii* [35,36,37,38,39,40,41], *Lachancea thermotolerans* [40,42,43], *Saccharomycodes ludwigii* [44], *Zygosaccharomyces rouxii* [44], *Hanseniaspora* spp. [45], *Starmerella bacillaris* (synonym *Candida zemplinina*) [46,47], *Metschnikowia pulcherrima* [48], and *Brettanomyces bruxellensis* [49] have been investigated for their ability to produce beers with enhanced aromatic profiles and low-alcohol or alcohol-free content.

Given its previously limited attention, substantial progress has recently been achieved in research on yeasts as probiotics [50,51,52,53,54,55,56,57,58,59]. According to the World Health Organization (WHO) and the Food and Agriculture Organization of the United Nations (FAO), probiotics are defined as live microorganisms that, when consumed in sufficient quantities, confer health benefits on the host [60,61]. The viability of probiotic candidates within the gastrointestinal tract is a critical factor, as they must withstand the challenges posed by digestive enzymes, gastric and bile salts, pH variations, and physiological temperature [54,56,57,58,59,62,63,64,65,66]. Furthermore, probiotic candidates must adhere to gut epithelial cells, resist antibiotics, inhibit pathogens, and be non-pathogenic, exhibiting no harmful effects [54,57,59,62,63,65]. Additionally, ease of production, prolonged shelf life when preserved, and genetic stability are considered essential for their success [53,54,55,56,57,58,64,65]. *Saccharomyces cerevisiae* var. *boulardii* is the most extensively studied probiotic yeast [58,67,68]. Additionally, *Sacch. cerevisiae* has been investigated for its beneficial probiotic properties [58,59]. Furthermore, other non-*Saccharomyces* genera, including *Hanseniaspora*, *Metschnikowia, Pichia*, *Torulaspora*, and *Starmerella*, are currently listed as genera with probiotic potential [56,57,58,59,69,70,71].

Beer has effectively delivered probiotics to consumers [72,73,74,75,76]. Recent research has explored the use of *Sacch. cerevisiae* var. *boulardii* in beer, particularly in craft varieties that maintain live cultures through unpasteurized and unfiltered production [77,78,79,80,81]. Additionally, using non-*Saccharomyces* yeasts represents a pioneering contribution to the brewing industry, which could lead to innovative beer production that blends tailored taste profiles with health-promoting properties [47].

This study addresses a critical gap by investigating the dual functionality of selected non-*Saccharomyces* and *Saccharomyces* strains, assessing their effectiveness as both probiotic agents and alternative starter cultures in craft beer production.

## 2. Materials and Methods

### 2.1. Yeast Strains and Growth Conditions

In this study, two non-*Saccharomyces* (MI120 and MI125 strains), which belonged to the Culture Collection of Microorganisms of the Faculty of Biotechnologies (UASMVB, România), were used. These strains were previously isolated from autochthonous Romanian grapes. The *Sacch. cerevisiae* BB06 strain (GenBank accession number: OL757483), with proven valuable probiotic properties, was used as a reference probiotic [58] for the preliminary probiotic characterization. The commercial *S. cerevisiae* US-05 strain (Fermentis by Lesaffre, Marquette-Lez-Lille, France) was used as a reference yeast for the brewing characterization. All yeast strains were preserved at −20 °C in YPSM broth [82] supplemented with 30% (*v*/*v*) glycerol (Scharlab S.L., Barcelona, Spain). The strains were streaked onto YPSM agar to reactivate them and incubated at 25 °C for 24 h. The yeast strains were then subcultured twice onto YPSM agar before use.

### 2.2. Molecular Identification

Yeast DNA was extracted using the ZR Fungal/Bacterial DNA Kit (Zymo Research, Irvine, CA, USA). The internal transcribed spacer (ITS) region was amplified with ITS1 and ITS4 primers (Biolegio B.V. Nijmegen, The Netherlands), as described by Dumitrache et al. [82]. Sequencing was performed at the Cellular and Molecular Immunological Applications (CEMIA, Larissa, Greece). The resulting sequences were subjected to bioinformatic analysis against the NCBI database using BLASTN (https://blast.ncbi.nlm.nih.gov/Blast.cgi/, accessed on 8 December 2024) to identify species based on percent identity. A phylogenetic analysis of the fungal groups was conducted using MEGA X version 10.1.8 [83], where ClustalW aligned ITS sequences and generated neighbor-joining (N-J) trees with 1000 bootstrap replications [84].

### 2.3. Probiotic Properties Characterization

#### 2.3.1. Resistance to Simulated Gastrointestinal Conditions

Yeast survival rate following simulated gastrointestinal exposure was assessed using a modified version of the method described by Diguță et al. [58]. Fresh yeast cultures were centrifuged at 2000× *g* for 5 min, washed twice with sterile PBS buffer (VWR International, Rosny-sous-Bois, France), and resuspended in the same PBS buffer. The yeast culture (approximately 10^7^ CFU·mL^−1^) was then inoculated into 10 mL of simulated gastric juice consisting of pepsin (0.3% *w*/*v,* Sigma Aldrich, Merck KGaA, Darmstadt, Germany) dissolved in sterile PBS buffer with the pH adjusted to 2.0 (with 1 N HCl, Altmann Analytik GmbH & Co. KG, Bernd Kraft, Duisburg, Germany), and then incubated under static anaerobic conditions at 37 °C for 3 h.

To evaluate the survival of yeast isolates in simulated intestinal conditions, a previously prepared yeast suspension (10^7^ CFU·mL^−1^) was mixed with a sterile PBS solution supplemented with 0.3% (*v*/*v*) bile salts (Sigma Aldrich, Merck KGaA, Darmstadt, Germany) and the pH adjusted to 8.0 (using 1 M NaOH, Altmann Analytik GmbH & Co. KG, Bernd Kraft, Duisburg, Germany). The mixture was then incubated at 37 °C for 4 h.

Across both trials, yeast cell viability was determined via the plate count method at baseline (0 h) and post-incubation (3 h and 4 h) in simulated gastrointestinal conditions. The survival rate was calculated according to the following formula:

$$\%\;viability=(\frac{log\;CFU\;Nt}{log\;CFU\;Ni})\times 100$$

Ni and Nt represent the log CFU·mL^−1^ at time 0 and at various time intervals, respectively.

#### 2.3.2. Antibacterial Activity

The antibacterial activity was evaluated using the cross-streaking method [85] against three Gram-positive pathogenic bacteria (*Bacillus cereus* ATCC 11778, *Listeria monocytogenes* ATCC 13932, and *Staphylococcus aureus* ATCC 33592) and five Gram-negative pathogenic bacteria (*Escherichia coli* ATCC 8739, *Pseudomonas aeruginosa* ATCC 15442, *Salmonella enterica* subsp. *enterica* serovar *Typhimurium* ATCC 14028, *Salmonella enterica* subsp. *enterica* serovar *Enteritidis* ATCC 13076, and *Serratia marcescens* ATCC 14756). Reference bacterial strains were procured from the American Type Culture Collection (ATCC, Manassas, VA, USA). Fresh yeast strains were centrally streaked in a single line on PDA plates (Alliance Bio Expertise, Guipry Messac, France). After 48 h of incubation at 25 °C, the pathogenic strains were streaked perpendicularly to the yeast streak. The plates were then incubated for an additional 24 h at 30 °C. Antibacterial activity was assessed by measuring the diameter of the clear zone of inhibition, which was recorded in millimeters (mm).

#### 2.3.3. Antibiotic Susceptibility

Yeast strain antibiotic susceptibility was assessed using the Kirby–Bauer disk diffusion method per CLSI [86]. Seven antibiotics from BioAnalyse (Ankara, Turkey), including ampicillin (AMP-10), chloramphenicol (C-30), erythromycin (E-15), fluconazole (FLU-25), itraconazole (ITR-10), ketoconazole (KCA-10), and miconazole (MCL-10), were tested. Overnight cultures (100 μL) were spread on YEPD agar plates and the antibiotic disks were applied. Following incubation, the inhibition zone diameters around the disks were measured with a ruler and classified as sensitive (>15 mm) or resistant (≤15 mm), according to Diguță et al. [58].

#### 2.3.4. Hemolytic Assay

Yeast strain hemolytic activity was determined following the protocol described by Diguță et al. [58]. Strains were streaked on blood agar plates (Oxoid, Basingstoke, Hampshire, UK). Following incubation, hemolysis activities were categorized as γ-hemolysis (no lysis, no color change), α-hemolysis (partial lysis, greenish hue), or β-hemolysis (complete lysis, clear zone around the colony).

#### 2.3.5. DPPH Free Radical Scavenging Activity

DPPH radical scavenging activity (RSA) was assessed following the protocol outlined by Brand-Williams et al. [87]. Briefly, 1 mL of cell-free supernatant (CFS) was mixed with 2 mL of a 0.2 mM DPPH solution (Sigma Aldrich, Merck KGaA, Darmstadt, Germany), freshly dissolved in absolute ethanol (VWR Chemicals, Leuven, Belgium). After vortexing for 2 min, the mixture was incubated in the dark at room temperature for 30 min. Absorbance was measured at 517 nm using a UV-1800 spectrophotometer (ChromTech, Minneapolis, MN, USA) with absolute ethanol as a blank and the DPPH solution alone as a control. The RSA percent was calculated as follows:

$$\%\;\mathrm{RSA}=\frac{Abs.control-Abs.sample}{Abs.control}\times 100$$

#### 2.3.6. Preservation of Yeast Cells

For long-term preservation, yeast cells were freeze-dried using the protocol outlined by Diguță et al. [58]. Fresh yeast cultures (24 h) underwent centrifugation at 2000× *g* for 5 min at 4 °C. The cell pellets were rinsed twice with PBS buffer and resuspended in 2 mL of a 5% (*w*/*v*) glucose solution (PanReac AppliChem, Darmstadt, Germany) as a cryoprotective agent. After being frozen at −20 °C overnight, the suspensions were freeze-dried in a FreeZone 6 freeze-dryer (Labconco, Kansas City, MO, USA) at −55 °C and 0.3 mbar for 4 h. The viability of the cells was evaluated before (expressed as log CFU Ni) and after (expressed as log CFU Nfd) the freeze-drying process using the plate count method. The survival rate of the yeast strains was calculated using the following formula:

$$\%\;viability=\frac{log\;CFU\;Nfd/mL}{log\;CFU\;Ni/mL}\times 100$$

### 2.4. Technological and Functional Yeast Screening

#### 2.4.1. Carbon Assimilation, Osmotic Tolerance, and CO_2_ Production

Fresh yeast strains were streaked onto Yeast Nitrogen Base (YNB) without amino acids (Sigma-Aldrich, St. Louis, MO, USA), supplemented with 2% (*w*/*v*) agar and either 2% (*w*/*v*) carbon sources (glucose, fructose, maltose, or sucrose) or 30% (*w*/*v*) glucose. All carbon sources were purchased from PanReac AppliChem (Darmstadt, Germany). Additionally, the strains were inoculated into Durham tubes containing YNB broth supplemented with 10% malt extract (Scharlab S.L., Barcelona, Spain) and incubated at 25 °C for 72 h to evaluate fermentation via CO_2_ accumulation in the Durham tubes.

#### 2.4.2. Ethanol and Low pH Tolerance

Yeast strains were streaked onto YPSM agar supplemented with ethanol (VWR Chemicals, Leuven, Belgium) at concentrations of 2.5%, 5%, or 7.5% (*v*/*v*) [85]. The plates were promptly sealed with Parafilm^®^ to minimize evaporation and incubated at 25 °C for 48 h. Ethanol tolerance was confirmed by visible yeast growth. Additionally, each strain (initial OD_600_ = 0.1 ± 0.010) was inoculated into YPSM broth with the pH adjusted to 3.0 (with 1 N HCl, Altmann Analytik GmbH & Co. KG, Bernd Kraft, Duisburg, Germany) and incubated at 18 °C for 24 h. Growth performance was determined by measuring absorbance at OD_600_ and interpreted as follows: no growth (−), OD_600_ = 0.1; weak growth (+), 0.1 < OD_600_ < 0.5; moderate growth (++), 0.5 < OD_600_ < 1.0; strong growth (+++), OD_600_ > 1.0.

#### 2.4.3. Acetic Acid Production

Yeast strains were inoculated onto Chalk agar plates and incubated at 25 °C for 7 days, as outlined by Sidari et al. [88]. The clear zone encircling the colonies indicated the rate of acetic acid production.

#### 2.4.4. H_2_S Production

Yeast strains were streaked onto BiGGY agar plates (Scharlab S.L., Barcelona, Spain) and incubated at 25 °C for 48 h. The color intensity of the colonies reflected the rate of H_2_S production, as described by Cordente et al. [89].

#### 2.4.5. Growth at Various Temperatures

Fresh yeast strains, starting at a concentration of 10^5^ CFU·mL^−1^, were inoculated into YPSM broth and subsequently incubated at 4 °C, 18 °C, and 37 °C for 24 h, following the protocols outlined by Diguță et al. [58] and Mogmenga et al. [59]. The ability of these strains to withstand a range of temperatures was evaluated after incubation using the plate count method.

#### 2.4.6. Enzymatic Activity

The enzymatic activities of yeast strains were assessed using the API ZYM system (Biomèrieux, Montalieu-Vercieu, France) according to the manufacturer’s guidelines. Following a 48 h incubation in YPSM broth at 28 °C, 60 µL of each yeast suspension was dispensed into the API ZYM strip wells and incubated at 37 °C for 4 h. Color changes in the wells, indicating positive enzymatic reactions on a scale from 0 (negative) to 5 (maximum intensity), were evaluated against the API ZYM color chart.

#### 2.4.7. Killer Toxin Assay

The killer/susceptible phenotype was assessed following the methodology outlined by Găgeanu et al. [90]. A specific medium was prepared consisting of 2% (*w*/*v*) glucose (PanReac AppliChem, Darmstadt, Germany), 1% (*w*/*v*) peptone (Merck KGaA, Darmstadt, Germany), 1% (*w*/*v*) yeast extract (Merck KGaA, Darmstadt, Germany), and 0.003% (*w*/*v*) methylene blue (Merck KGaA, Darmstadt, Germany) dissolved in sodium citrate buffer, with the pH adjusted to 4.8 using 1 N HCl. *Sacch. cerevisiae* strain 17/17 was used as the susceptible reference strain, while *Sacch. cerevisiae* strain SMR4 served as the killer reference strain. Each reference strain was evenly spread on the surface of the culture medium, after which the yeast strains under investigation were streaked onto the same medium for testing. These plates were then incubated for 3 days at 18 °C. A blue-stained killing zone at the spot indicated the sensitive phenotype, while a blue-stained zone encircled by an inhibition halo around the cells denoted the killer phenotype. Yeast cells exhibiting no reaction were classified as having a neutral phenotype.

### 2.5. Pilot-Scale Fermentations

#### 2.5.1. Inoculum Preparation

To initiate beer fermentation, yeast strains were propagated aerobically in YPSM broth at 25 °C for 48 h and then centrifuged at 3000 rpm for 10 min at 4 °C. The cell pellet was washed twice with sterile PBS buffer (VWR International, Rosny-sous-Bois, France) and used as the starter, with its viable cell concentration determined via the plate count method. Commercial *Sacch. cerevisiae* US-05, prepared according to the supplier’s guidelines, served as the reference strain.

#### 2.5.2. Beer Wort Preparation and Fermentation Conditions

The two types of malts (Pale Ale Malt and CARAPILS^®^) were purchased from Weyermann Specialty Malting (Bamberg, Germany), with the main physico–chemical and technological characteristics supplied by the producer.

The Cascade T90 hops (purchased from a local distributor in Bucharest (Romania) were used for wort boiling and added at 0, 5, 15, and 60 min.

The brewing experiments were performed in the Pilot Laboratory for Obtaining Fermented Drinks, part of the Faculty of Land Reclamation and Environmental Engineering at the UASMV Bucharest. The technological diagrams were as follows: the mashing was performed using a blend of Pale Ale and CARAPILS^®^ malts, applying an infusion diagram typically used for India Pale Ale (IPA) beers, followed by 60 min of boiling with hop pellets. At the end of boiling, the wort was transferred to a whirlpool vessel for clarification, and the clarified wort was then cooled using the system’s plate heat exchanger. Subsequently, it was distributed into glass fermentation vessels (5 L per variant) in triplicate. The commercial *Sacch. cerevesiae* US-05 producer indicated pitching and fermentation technological parameters. The primary fermentation process lasted for 7 days, at a controlled temperature of 18 °C, followed by a transfer into 330 mL brown glass bottles, along with 0.6% (*w*/*v*) sugar (purchased from a local distributor in Bucharest, Romania) per bottle to initiate secondary fermentation, which was carried out in the bottles for 14 days at 18 °C. After this period, the bottles were stored in a refrigerator until analysis. All beer samples were produced using the same technological diagram.

#### 2.5.3. Physico-Chemical Analyses of Wort and Beer

##### Ethanol Content and Original Extract of Beer

The determination of alcohol in beer samples was performed through densitometry using a hydrostatic balance. The beer samples were prepared by degassing filtration through dry filter paper. An electronic distilling unit (Super D.E.E., Gibertini, Milano, Italy) and a hydrostatic balance (Densi Alcomat, Gibertini, Milano, Italy) were used as equipment. The results for alcohol were expressed as % (*v*/*v*), to two decimal places. The original beer extract was calculated based on the specific gravity of the beer, as well as alcoholic distillate and beer residue after the distillation step, according to the methods described by Analytica-EBC (2005). The results for the original extract were expressed as % Plato, to one decimal place [91].

##### Bitterness of Beer

The bitterness of beer samples was measured using the UV spectrophotometric method using a spectrophotometer (PhotoLAB 7600 UV-VIS, WTW, Grammetal, Germany) (absorbance measurement at 275 nm against pure iso-octane, purchased from Merck KGaA, Darmstadt, Germany) after acidification with iso-octane. The results for bitterness were expressed as BU value to the nearest whole number [92].

##### Total Acidity

Total acidity in wort and beer samples was determined according to the method described by the Romanian standard SR 13355-6:2000 [93] by measuring the amount of sodium hydroxide (Altmann Analytik GmbH & Co. KG, Bernd Kraft, Duisburg, Germany) until the neutralization endpoint was reached. The results for color were expressed as mL NaOH 1 N per 100 mL beer/wort to two decimal places.

##### Color of Beer

The determination of color in beer samples was performed using the spectrophotometric method according to the methods described by Analytica-EBC (2005) using a spectrophotometer (PhotoLAB 7600 UV-VIS, WTW, Grammetal, Germany). The absorbance of the beer was measured at 430 nm using a 10 mL cell [94,95]. The results for color were expressed as EBC units in two decimal places.

##### Soluble Dry Matter in Wort and Sugar Determination in Wort and Beer

The soluble dry matter (Brix value) for wort was determined by refractometry using an Abbemat 550 refractometer (Anton Paar OptoTec GmbH, Seelze-Letter, Germany). The results were expressed in g/100 g.

An HPLC method with refractive index detection (HPLC-RID) determined sugar in the wort and beer samples. The sample preparation procedure for beer was as follows: the sample was filtered through a regenerated cellulose 0.45 mm filter and then injected into the HPLC system. For the wort samples, 5 mL was measured into a 50 mL volumetric flask, over which water was added and ultrasonicated. After cooling, the clarification solutions (Carrez I and Carrez II, Carl Roth GmbH, Karlsruhe, Germany) were added and then shaken; further, the solution was brought with water to the mark. After the separation, samples were filtered through a regenerated cellulose 0.45 mm filter and injected into the HPLC system. The following instruments were used for HPLC analysis: a Shimadzu HPLC instrument (Shimadzu Corporation, Tokyo, Japan) with DGU-405 degassing unit; LC-40D pump; SIL-40 autosampler/injector; CTO-40C column oven; RID-20A refractive index detector; Shimadzu LabSolutions software, version 5.110; Phenomenex Luna Omega SUGAR column, 150 mm x 46 mm. The elution was performed using acetonitrile/water mixture (82:18) as the mobile phase at a flow rate of 0.8 mL/min and a temperature of 25 °C. The refractive index detector was maintained at 40 °C, and the injection volume was 10 µL. The quantitative determination was performed on external standards (ribose, xylose, arabiosis, fructose, mannose, glucose, galactose, sucrose, turanose, maltose, trehalose, lactose, melesitose, maltotriose, and raffinose) within the concentration range from 0.010% to 1%. The results were expressed as a percentage of each sugar’s mass/volume (g/100 mL). The external sugar standards were purchased from LGC Standards (Dr. Ehrenstorfer, Teddington, UK) and Sigma-Aldrich (Merck KGaA, Darmstadt, Germany).

##### Real Degree of Fermentation of Beer

For a more detailed assessment of the performance of the brewing yeast, the real degree of fermentation (RDF), also called attenuation, was calculated for the experimental beer samples. As a technological parameter, the RDF represents the extract ratio initially present in the wort and transformed into alcohol by the brewing yeast [96]. RDF is calculated using the following formula:

$$RDF=100\cdot \frac{2.0665\cdot A}{2.0665\cdot A+ER}\%$$

where

A is the alcohol % (m/m);
ER is the real extract, % Plato.

##### Evaluation of the Total Polyphenol Content

The total polyphenolic content of the beer samples was assessed using the Folin–Ciocalteu method. In short, 110 µL of 10% Folin–Ciocalteu reagent (Sigma Aldrich, Merck KGaA, Darmstadt, Germany) was combined with 70 µL of 20% Na_2_CO_3_ solution (J.T. Baker, Mallinckrodt Baker, Phillipsburg, NJ, USA) and 20 µL of the beer samples. This mixture was incubated in darkness at room temperature for 30 min, after which the absorbance was recorded at 765 nm. A calibration curve of Gallic acid (0.10–200 μg·mL^−1^, Sigma Aldrich, Merck KGaA, Darmstadt, Germany) was utilized, and the total phenolic content of the beer samples was represented as μg Gallic acid equivalents per mL of sample (μg GAE·mL^−1^).

##### DPPH Free Radical Scavenging Activity of Beer Samples

The DPPH radical scavenging activity (RSA) was evaluated according to the protocol outlined in Section 2.3.5.

#### 2.5.4. Assessment of Yeast Cell Survival in Beer Samples Following a 2-Month Storage Duration

The viability of yeast cells in craft beer was determined after 2 months of storage post-bottling via the plate count method.

#### 2.5.5. Sensorial Analysis

The sensory characteristics of the experimental beer samples, which were anonymous, were evaluated by a panel of 20 tasters, comprising men and women aged between 20 and 50. The jury was informed of the tasting details, the type, and the number of samples to be analyzed and was aware of possible alcohol ingestion. The following quality indicators were rated: foam, smell, taste, color, aftertaste, acidity, bitterness, and body. Beer samples were evaluated using a 0–5 scoring scale, with 0 indicating ‘altered’ and 5 representing ‘excellent’. Before testing, the beer samples were kept at room temperature for 30 min. The research complied with the Declaration of Helsinki, with the protocol approved by the UASMV Bucharest Ethics Committee (Proceedings no. 1674, dated 12 March 2025). The sensory indicators analyzed were plotted in a radar chart [97].

### 2.6. Statistical Analysis

All experiments were conducted independently in triplicate, and the results are expressed as means ± standard deviation. A one-way analysis of variance (ANOVA) was performed using GraphPad Prism Software version 10.4.1 to assess statistical significance. Subsequently, Tukey’s test was applied to determine significant differences among the means, with a significance level of *p* < 0.05.

## 3. Results

### 3.1. Molecular Identification of Yeast Strains Based on ITS rDNA Sequencing 

**Figure 1:**
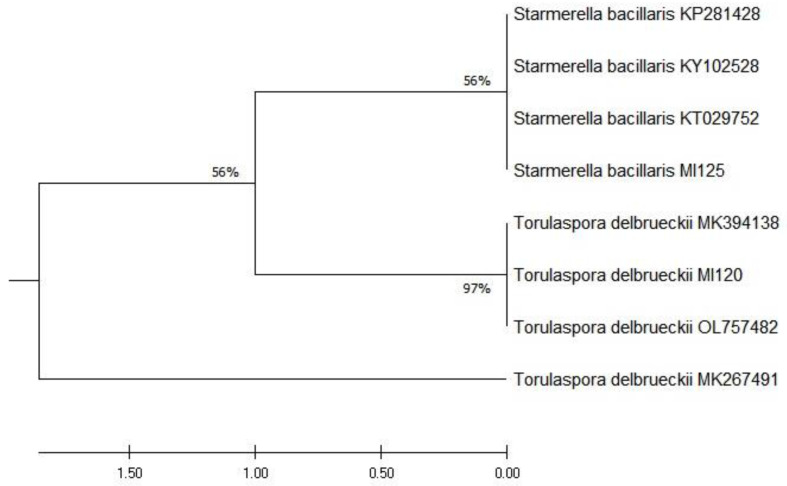
Phylogenetic tree generated using MEGA X software from 5.8S rDNA sequences, aligned with ClustalW and analyzed via UPGMA clustering.

The yeast strains used in this investigation were isolated from grapes and preserved in the Microorganisms Collection at the Faculty of Biotechnologies, UASMV Bucharest. The species identification was achieved by comparing their 5.8S rDNA sequences to those deposited in the NCBI database. The 5.8S rDNA sequence of strain MI120, archived under GenBank accession no. PV202278, showed 99.48–100% identity with 100 *Torulaspora delbrueckii* sequences in the NCBI database. Similarly, the 5.8S rDNA sequence of strain MI125 was identified as *Starmerella bacillaris*, accessible under GenBank accession no. PV202279. A comparative evaluation of these 5.8S rDNA sequences against reference strains was conducted using MEGA X software version 10.1.8, with ClustalW for sequence alignment and phylogenetic tree construction, as shown in Figure 1.

### 3.2. Assessment of Yeast Strains as Potential Probiotics

#### 3.2.1. Resistance in Simulated Digestive Conditions

**Figure 2:**
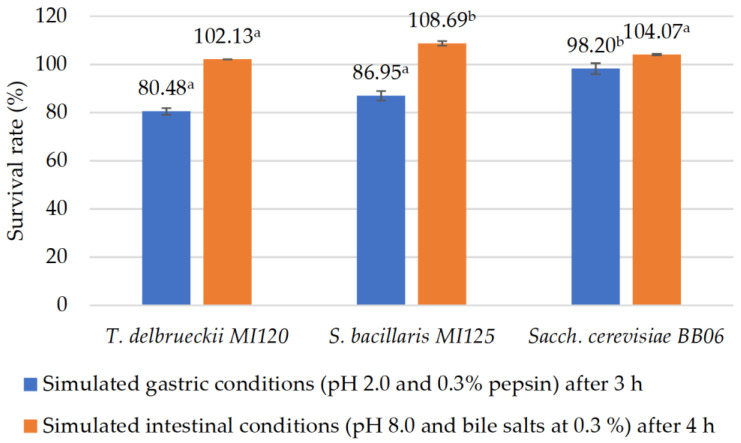
In vitro evaluation of probiotic properties of yeast strains under simulated gastrointestinal conditions. Results are reported as means ± standard deviations (SDs) based on three experiments, with significant variations (*p* < 0.05) among strains quantified by the Tukey B test from one-way analysis of variance (ANOVA) and marked by distinct letters.

All the strains started with a mean initial cell density of 7 log CFU·mL^−1^. The yeast strains were assessed for survival rates under simulated gastric (pH 2.0, 0.3% pepsin) and intestinal (pH 8.0, 0.3% bile salts) conditions, as shown in Figure 2.

Following three hours of incubation under simulated gastric conditions, all the yeast strains showed a high tolerance, with survival rates ranging from 80.48% to 98.20%, as shown in Figure 2.

Under simulated intestinal conditions, a moderate increase in viable cell counts (0.15–0.61 log) was observed after four hours of exposure (Figure 2).

#### 3.2.2. Antibacterial Properties

The antibacterial properties of the yeast strains were assessed against a panel of eight pathogenic bacteria, as reported in Table 1. *S. bacillaris* MI125 demonstrated greater antibacterial efficacy against all the tested pathogens than the probiotic strain *Sacch. cerevisiae BB06.* In contrast, *T. delbrueckii* MI120 displayed the least antibacterial activity, limited to *Bacillus cereus* and *Serratia marcescens.*

#### 3.2.3. Assessment of the Antibiotic Sensitivity of Yeast Strains

The antibiotic resistance profile of the yeast strains against three antibacterial and four antifungal antibiotics was examined (Table 2). *S. bacillaris* MI125 and *Sacch. cerevisiae* BB06 exhibited resistance to all the tested antibiotics, whereas *T. delbrueckii* MI120 resisted the antibacterial agents yet demonstrated susceptibility to fluconazole, ketoconazole, and miconazole.

All the tested yeast strains showed promise as probiotic supplements for preventing and managing antibiotic-associated diarrhea, emphasizing their resilience against antibiotic treatment and their favorable safety profiles.

#### 3.2.4. Hemolysis Assay

Our investigation revealed that each yeast strain exhibited γ-hemolysis (no detectable greenish or clear zones around the colonies), enhancing their safety profiles for potential probiotic applications.

#### 3.2.5. Antioxidant Properties

**Figure 3:**
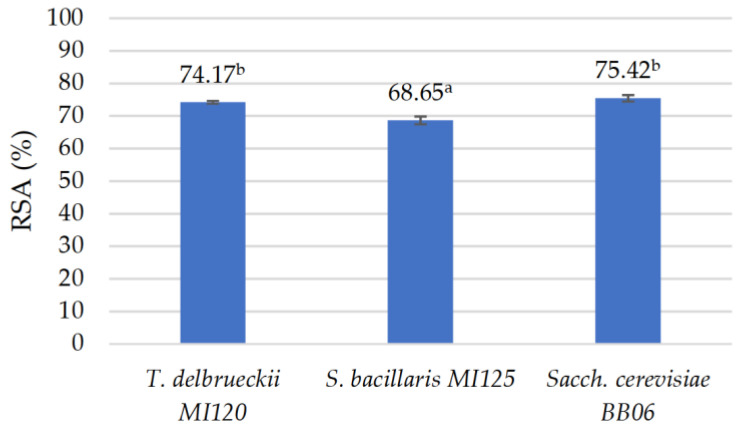
Evaluation of antioxidant properties of yeast cell-free supernatant via the DPPH assay. Results are reported as means ± standard deviations (SDs) based on three experiments, with significant variations (*p* < 0.05) among strains quantified by the Tukey B test from one-way analysis of variance (ANOVA) and marked by distinct letters.

The antioxidant activity of the yeast CFS was assessed using the DPPH assay. The results confirmed that all the strains possessed a strong antioxidant capacity, exceeding 60%, as illustrated in Figure 3. *T. delbrueckii* MI120 displayed an RSA rate of 74.17 ± 0.48%, closely aligning with the reference *Sacch. cerevisiae* BB06 at 75.42 ± 0.99%, and significantly surpassing *S. bacillaris* MI125 at 68.65 ± 1.18%.

#### 3.2.6. Preservation of Probiotic Yeast Strains via Freeze-Drying Method

**Figure 4:**
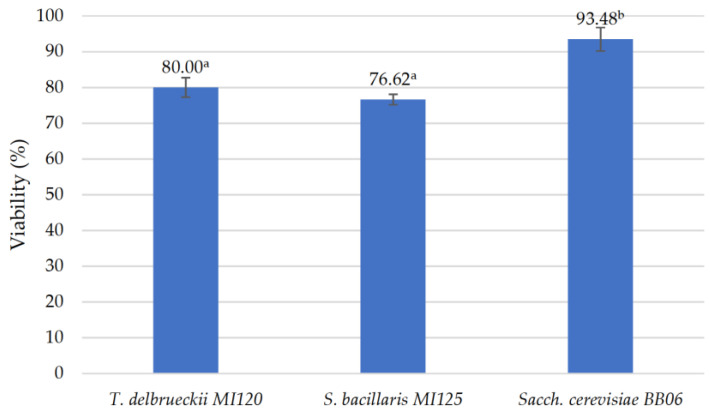
Viability percentage of freeze-dried yeast strains. Results are reported as means ± standard deviations (SDs) based on three experiments, with significant variations (*p* < 0.05) among strains quantified by the Tukey B test from one-way analysis of variance (ANOVA) and marked by distinct letters.

The viability percentages of the freeze-dried yeast strains underscored that *Sacch. cerevisiae* BB06 exhibited the highest resilience when preserved with glucose as the cryoprotectant, achieving a survival rate of 93.48 ± 3.29%. It significantly surpassed *T. delbrueckii* MI120 at 80.00 ± 2.74% and *S. bacillaris* MI125 at 76.62 ± 1.47%, as shown in Figure 4.

### 3.3. In Vitro Assessment of Yeast Strains for Brewing Potential

Regarding carbon assimilation, all yeast the strains were grown on a YNB medium supplemented with glucose, fructose, maltose, and sucrose (Table 3). The non-*Saccharomyces* strains (*T. delbrueckii* MI120 and *S. bacillaris* MI125) showed no CO_2_ production (empty Durham tubes), while *S. cerevisiae* (BB06 and US-05 strains) produced large amounts of CO_2_ (full Durham tubes). All the strains displayed a strong growth capacity at pH 3.0. All the strains exhibited high growth in culture media containing 0–5% ethanol. *Sacch. cerevisiae* BB06, and commercial *Sacch. cerevisiae* US-05 demonstrated tolerance to 7.5% ethanol, in contrast to *S. bacillaris* MI125 (Table 3). All the strains consistently produced acetic acid. Hydrogen sulfide (H_2_S) production, an undesirable metabolite associated with off-flavors, was evaluated using a BiGGY agar medium. The results indicated that all the yeast strains produced moderate H_2_S levels, as evidenced by their brown-colored biomass (Table 3).

According to Table 3, *S. bacillaris* MI125, *T. delbrueckii* MI120, and *Sacch. cerevisiae* BB06 demonstrated a neutral phenotype against both *Sacch. cerevisiae* control strains. However, commercial *Sacch. cerevisiae* US-05 demonstrated a sensitive phenotype against the killer *S. cerevisiae* strain.

**Figure 5:**
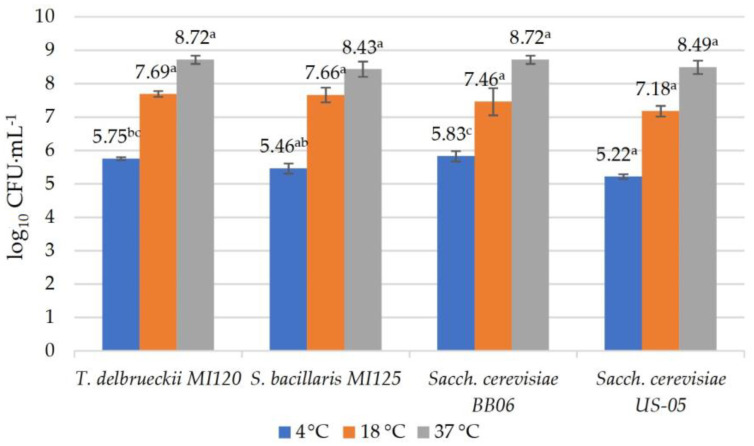
Temperature tolerance of yeast strains. Results are reported as means ± standard deviations (SDs) based on three experiments, with significant variations (*p* < 0.05) among strains quantified by the Tukey B test from one-way analysis of variance (ANOVA) and marked by distinct letters.

All the yeast strains displayed slow growth at 4 °C (beer maturation temperature) with 0.1–0.4 log CFU·mL^−1^. They displayed strong growth at 18 °C (fermentation temperature), between 1.98 and 2.66 log CFU·mL^−1^, and pronounced growth at 37 °C (body temperature), between 3.14 and 3.46 log CFU·mL^−1^ (Figure 5).

A semi-quantitative analysis of yeast strain enzymatic activities was conducted using the API ZYM test, which covers 20 enzymes across the categories of esterases, glycoside hydrolases, phosphatases, and proteases; the outcomes are reported in Table 4. *T. delbrueckii* MI120 showed a range of enzymatic activities, from low to high, including alkaline phosphatase, β-glucuronidase, cystine arylamidase, acid phosphatase, naphthol-AS-BI-phosphohydrolase, α-galactosidase, valine arylamidase, lipase esterase (C8), esterase (C4), and leucine arylamidase. *S. bacillaris* MI125 exhibited esterases (including esterase (C4) and lipase esterase (C8)), glycoside hydrolases (β-glucosidase), proteases (cystine arylamidase, valine arylamidase, and leucine arylamidase), and phosphatases (alkaline phosphatase, acid phosphatase, and naphthol-AS-BI-phosphohydrolase). The API ZYM enzymatic profile of *Sacch. cerevisiae* BB06 aligned with that of *Sacch. cerevisiae* US-05 (commercial yeast), characterized by moderate esterases (esterase (C4) and lipase esterase (C8)), proteases (cystine arylamidase and valine arylamidase), phosphatases (acid phosphatase and naphthol-AS-BI-phosphohydrolase), and weak β-glucuronidase activity. In contrast, *Sacch. cerevisiae* BB06 typically showed weak or high activity for alkaline phosphatase and leucine arylamidase. Meanwhile, *Sacch. cerevisiae* US-05 presented other glycoside hydrolases, such as α-galactosidase and β-galactosidase, and high activity for α-glucosidase.

### 3.4. Characterization of Beer Samples

#### 3.4.1. Physico-Chemical and Technological Characterization of Beer Fermented with Selected Yeast Strains

Table 5 shows the physico-chemical and technological characteristics of the pilot-scale beer samples.

While the mashing process for the experimental beer samples was similar, the original extract (Ep) of the samples ranged from 10.90% to 11.37%. The highest value was recorded for the beer sample produced with the commercial *Sacch. cerevisiae* US-05 (11.37 %P), closely followed by the sample produced with the *T. delbrueckii* MI120 strain (11.08 %P). Regarding the alcohol content, it ranged between 3.36% and 4.26% (*v*/*v*), showing a similar trend to the Ep; thus, the highest value was the beer sample produced with the commercial *Sacch. cerevisiae* US-05 (4.26% *v*/*v*), followed by the sample produced with the *T. delbrueckii* MI120 strain (4.21% *v*/*v*).

The color of the beer samples varied between 9 and 9.78 EBC, while the difference in bitterness among the experimental beer variants was negligible. The total acidity of the beer samples ranged between 1.6 and 2.23 mL NaOH 1 N per 100 mL of beer, with the highest value for the beer fermented with *S. bacillaris* MI125 (2.23 mL NaOH 1 N per 100 mL of beer).

The sugar spectrum for the wort and beer samples is given in Table 6.

Regarding the ability to ferment sugars and produce beer, it was observed that all the yeast strains demonstrated a genuine capacity for wort fermentation in the brewing industry. The RDF of the beer samples varied between 48.28% and 59.17%. The highest capacity to convert the extract initially present in the wort into alcohol (RDF 59.17%) was recorded for the *T. delbrueckii* MI120 strain, which exhibited a superior fermentation capacity compared to the commercial *Sacch. cerevisiae* US-05 (RDF 58.39%). Regarding the behavior of the yeasts with the spectrum and evolution of sugars, it was noted that the same wort used for the inoculation of all the beer samples, except for the beer produced with the *T. delbrueckii* MI120 strain, retained some fructose. Additionally, all the yeast strains completely metabolized glucose and sucrose. Nevertheless, the concentration of fructose in the beer samples was at LoQ levels (LoQ = 0.01 g/100 mL), varying between 0.01 and 0.012 g/100 mL. The other sugars identified in the beer samples were maltose (ranging from 0.019 to 0.046 g/100 mL; however, it was not detected in the beer produced with the commercial *Sacch. cerevisiae* US-05) and maltotriose (0.043 g/100 mL for the beer fermented with the *T. delbrueckii* MI120 strain).

The total polyphenol content (TPC) was expressed as μg Gallic acid equivalents per mL of sample (μg GAE·mL^−1^) (Table 7).

The beers fermented with *T. delbrueckii* MI120 displayed an elevated TPC of 96.02 ± 4.57 μg GAE·mL^−1^, close to the reference strain *Sacch. cerevisiae* US-05, which recorded 93.65 ± 2.55 μg GAE·mL^−1^. In contrast, those fermented with *S. bacillaris* MI125 and *Sacch. cerevisiae* BB06 exhibited lower TPC values of 76.59 ± 3.15 and 74.97 ± 1.69 μg GAE·mL^−1^, respectively. All the beer samples exhibited strong antioxidant activity (Table 7).

#### 3.4.2. Assessment of Yeast Cell Viability in Craft Beer Following 2 Months of Storage

Yeast cell survival in craft beer was assessed following two months of post-bottling storage. The findings indicated a decrease in survival across all the tested strains, with viable cell counts ranging from approximately 2 to 3 log CFU·mL^−1^, compared to an initial inoculum of about 7 log CFU·mL^−1^.

#### 3.4.3. Sensorial Analysis of Beer Samples

**Figure 6:**
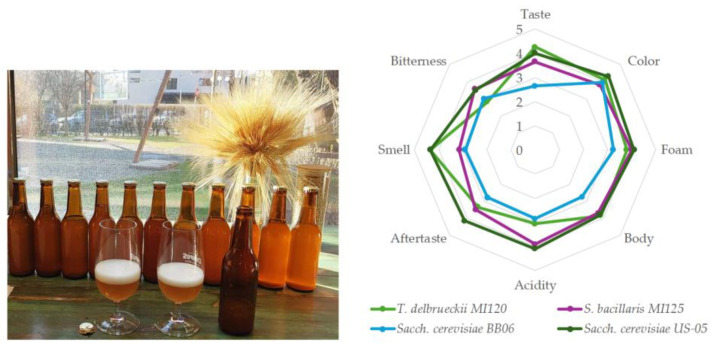
Radar charts of sensorial characteristics of beer samples.

The sensory analysis of the beers fermented with the distinct yeast strains indicated significant variations in taste, aroma, bitterness, and additional key attributes, as graphically represented in Figure 6.

*T. delbrueckii* MI120 and *Sacch. cerevisiae* US-05 received the highest ratings for taste (4.25 and 4.00) and aroma (4.35 and 4.35), suggesting an enhanced production of desirable flavor and aromatic compounds. In contrast, *S bacillaris* MI125 exhibited lower ratings for taste (3.65) and aroma (3.15), while *Sacch. cerevisiae* BB06 yielded less favorable sensory profiles (2.65 taste and 2.90 aroma). *Sacch. cerevisiae* US-05 excelled in terms of color (4.30) and foam stability (4.10), whereas *S. bacillaris* MI125 demonstrated strong foam retention (4.00), highlighting its potential for sustaining stability. *T. delbrueckii* MI120 (3.85) and *Sacch. cerevisiae* US-05 (3.80) delivered the most substantial body, contributing to a well-rounded mouthfeel, whereas *Sacch. cerevisiae* BB06 (2.75) resulted in the weakest body, suggesting a thinner beer profile. *Sacch. cerevisiae* US-05 exhibited the highest acidity (4.10), contributing to a refreshing note, followed by *S. bacillaris* MI125 (3.90), which may have enhanced its tartness. The highest recorded bitterness was found in *S. bacillaris* MI125 (3.55) and *Sacch. cerevisiae* US-05 (3.50), potentially intensifying the hop bitterness. *Sacch. cerevisiae* US-05 (4.15) was found to have the most notable aftertaste, followed by *S. bacillaris* MI125 (3.50) and *T. delbrueckii* MI120 (3.35), while *Sacch. cerevisiae* BB06 (2.80) had the least significant level of bitterness.

## 4. Discussion

Current trends in craft beer production focus on developing beers with enhanced nutritional value, health benefits, and distinct sensory characteristics to meet consumer preferences.

A compelling aspect of incorporating these yeasts as probiotics is the potential health benefits of consuming viable cultures in unpasteurized beer [72,73,74,75,76]. *S. cerevisiae* var. *boulardii* is considered safe and is exclusively marketed as a probiotic [77,78,79,80,81]. This trend aligns with the rising demand for sustainable probiotic alternatives, particularly concerning the challenges such as antibiotic resistance and evolving consumer preferences for innovative, health-promoting fermented products [56,57,58,59,69,70,71].

To our knowledge, this is the first comprehensive study investigating non-*Saccharomyces* and *Saccharomyces cerevisiae* strains for their dual functionality as probiotic agents and starter cultures in developing innovative craft beers that integrate distinctive sensory characteristics with potential health-promoting benefits.

Yeast probiotic potential is commonly assessed through in vitro standardized tests, examining species identification, gastrointestinal survival (body temperature, low pH, digestive enzymes, and bile salts), adhesion to gut epithelial cells, antibiotic resistance, pathogen suppression, and non-pathogenicity [54,56,57,58,59,63,64,65,66]. Furthermore, industrial scalability and prolonged shelf stability are essential for commercial applications [50,53,54,55,56,57,64,65,69]. In our study, 5.8S rDNA sequencing was used for species identification, confirming the MI120 strain as *T. delbrueckii* and the MI125 strain as *S. bacillaris*. Moreover, the interest in the probiotic potential of these non-*Saccharomyces* yeasts has grown, driven by their functional versatility and adaptability in diverse applications [58,70,98,99].

Our in vitro tests showed that all the yeast strains strongly survived simulated gastric conditions (pH 2.0, 0.3% pepsin) and simulated intestinal fluids (pH 8.0, 0.3% bile salts), satisfying a key criterion for probiotic potential. Our findings align with those reported in other studies. In another study, Diguță et al. [58] demonstrated that *Sacch. cerevisiae* BB06 and *T. delbrueckii* MT07 strains exhibited higher or comparable viability to *Sacch. boulardii* following 3 h of exposure to simulated gastric conditions. Moreover, these yeast strains demonstrated a notable tolerance to simulated intestinal conditions (0.3% bile salts) [58]. *Saccharomyces cerevisiae* strains, isolated from Rabilé beer, showed high endurance during exposure to simulated gastrointestinal environments [59]. According to Shen et al. [70], *S. bacillaris* CC-PT4, isolated from grape peel, tolerates gastric and intestinal fluids.

In this study, all the yeast strains tolerated 37 °C, the human body temperature. Our data are consistent with the results obtained by Mogmenga et al. [59]. The *S. bacillaris* CC-PT4 strain exhibits robust growth at 37 °C, as reported by Shen et al. [70]. Conversely, in contrast to the findings of Staniszewski and Kordowska-Wiater [99], none of the *S. bacillaris* isolates demonstrated the capacity to grow at human body temperature.

Our investigation revealed that all the yeast strains tested demonstrated γ-hemolysis, complying with EFSA safety criteria and consistent with the evidence from existing studies. The susceptibility assays showed that all the yeast strains exhibited resistance to antibacterial antibiotics. However, *T. delbrueckii* MI120 was sensitive to common antifungal agents.

Moreover, these strains exhibited notable antioxidant attributes, with values greater than 68%. Our results surpassed the values obtained by Diguță et al. [58] and Mogmenga et al. [59].

Among the most valued attributes of probiotics is their ability to suppress pathogens. *S. bacillaris* MI125 exhibited pronounced and broader antibacterial activity compared to the reference *Sacch. cerevisiae* BB06. Our results concur with the observations of Diguță et al. [58]. In another study, *S. bacillaris* exhibited anti-MRSA activity against antibiotic-resistant *Staphylococcus aureus* [70].

Due to their high tolerance to freeze-drying, the yeast strains are suitable for commercial applications in freeze-dried formulations [58]. Our yeast strains exhibited markedly higher survival rates compared to the findings obtained by Nicolae et al. [100].

The craft beer segment of the brewing industry has recently experienced a notable shift toward using non-*Saccharomyces* yeasts. This change is driven mainly by consumer preferences for distinctive, premium beers and a broader variety of available craft beers. Historically, *Saccharomyces* yeast has reigned supreme in brewing, prized for its reliable fermentation performance and ability to uphold beer quality [12,13,14,15]. However, due to their wide-ranging metabolic abilities, *S. bacillaris* and *T. delbrueckii* are gaining attention as compelling options for the production of craft beers with unique sensory characteristics [35,36,37,38,39,40,41,46,47]. The phenotypic tests encompassed an evaluation of growth across various fermentation conditions. In our study, all the yeast strains exhibited metabolic adaptability through their capacity to process all the assessed carbon sources, a characteristic of considerable significance to beer production. Yeasts, during fermentation, synthesize several sulfur compounds, including H_2_S, which can unfavorably impact beer by changing its flavor profile or concealing other compounds [89]. Our semi-quantitative evaluations showed that all the tested yeast strains, including the commercial strain, exhibited moderate H_2_S production levels. Our yeast strains, derived from grapes, demonstrated an elevated tolerance to ethanol levels of up to 5%. However, only *T. delbrueckii* MI120 and the *Saccharomyces* strains could endure 7.5% ethanol. The killer trait is particularly relevant in the winemaking and brewing industries, as it influences microbial competition, fermentation dominance, and spoilage prevention [39,88,89]. In our investigation, the yeast strains exhibited a neutral phenotype, whilst commercial *Sacch. cerevisiae* US-05 displayed a sensitive phenotype. *S. bacillaris,* commonly found in grapes and wines, is increasingly valued in wine fermentation for its metabolic behavior, which refines must and wine characteristics through key metabolites. For winemakers, *S. bacillaris* co-fermented with *Sacch. cerevisiae* strengthens wine stability, improves sensory qualities, and decreases ethanol content due to *S. bacillaris*’s limited ethanol production [46]. Numerous investigations suggest that *T. delbrueckii*’s strain-dependent fermentation process produces fruity beers with higher alcohols and esters at a slower rate than *Sacch. cerevisiae* [30,36]. This is due to incomplete sugar wort fermentation, which leads to ethanol levels ranging from 0.8 to 4% (*v*/*v*) [37,38]. This suggests that certain strains may be considered suitable for brewing purposes. Canonico et al. [101] explored *T. delbrueckii* in beer production, producing low-alcohol (2.66% *v*/*v*) beers with unique aromatic profiles; in mixed fermentations with *Sacch. cerevisiae,* it enhanced fruity esters and higher alcohols, in contrast to its lower higher-alcohol yield in winemaking. Similarly, Michel et al. [37] explored various *T. delbrueckii* strains from different habitats, finding one that produced fruity and floral aromas (2-phenylethanol and amyl alcohols), as well as two others suitable for low-alcohol beer due to their inability to ferment maltose and maltotriose, while retaining good flavor.

Our study demonstrated that all the yeast strains displayed elevated growth at the fermentation temperature (18 °C) and the body temperature (37 °C). Fermentation at lower temperatures enhances the beverage’s sensory properties via greater volatile compound formation [102,103], while high viability at 37 °C underpins their probiotic potential [58].

Throughout beer wort fermentation, yeast strains generate higher alcohols and esters as volatile compounds dependent on their enzymatic profiles, playing a substantial role in defining the sensory properties of the resulting beer [104].

In addition, the values of total acidity, bitterness, and color were similar to those of the beers produced from barley malts. These findings align with the quality parameters of blonde beers according to the Romanian standard in force for beer [105] and are consistent with the findings of other researchers [14,106,107,108].

The alcohol content for the beers fermented with *T. delbrueckii* MI120 and *Sacch. cerevisiae* BB06 aligns with the results observed for craft beer produced and analyzed by other authors, including those made using non-*Saccharomyces* yeast [30,36,37,101]. The higher content of fermentable sugars in wort was noticed for maltose, followed by maltotriose. At the same time, fructose was the compound with the lowest concentration, which aligns with the relevant literature references [109,110,111]. However, the RDF for the commercial *Sacch. cerevisiae* US-05 during the experiments performed was lower than the attenuation reported by the relevant literature (78–82%), which is considered medium [112], as well as those expected from the manufacturer’s specifications for attenuation. Other authors have confirmed the overall evolution of fermentable sugars through beer production [109,110,111,113]. For example, based on the results registered both for the content of individual sugars as well as for the RDF, it was found that simple sugars can be fully fermented by yeasts (*Saccharomyces* and non-*Saccharomyces*) used for the fermentation of brewing wort, therefore recommending these strains for craft as well as for industrial beer production. A noteworthy finding, which is highly relevant for practical applications in the brewing industry, is that no special technological conditions (e.g., wort composition or supplementation, specific levels for technical parameters such as pH or temperature) are required by the studied *Saccharomyces* and non-*Saccharomyces* yeast strains used, thus recommending these strains for successful use for brewing in an operational environment.

The antioxidant properties of beer are primarily attributed to phenolic compounds. In this study, the beers produced by *T. delbrueckii* MI120 and the commercial *Sacch. cerevisiae* US-05, characterized by a higher total phenolic content, displayed elevated antioxidant activity. However, *S. bacillaris* MI125, despite a reduced TPC, achieved a similar antioxidant activity level.

The application in practice confronts technical challenges, as the probiotic viability must be preserved during storage processes. Nevertheless, following a two-month post-bottling storage duration, the count of viable cells exhibited a significant reduction, decreasing by 2–3 log CFU·mL⁻^1^.

Multiple studies have highlighted the potential of *Saccharomyces cerevisiae* var. *boulardii* in producing functional beers. For instance, Senkarcinova et al. [80] and Ramirez-Cota et al. [81] showed that *Sacch. cerevisiae* var. *boulardii*’s ability to tolerate ethanol allowed for low-alcohol and alcohol-free craft beers with probiotic properties. Mulero-Cerezo et al. [79] showed that *Sacch. cerevisiae* var. *boulardii*, as the sole starter, produced a beer with increased antioxidants, reduced alcohol, similar sensory qualities, and higher yeast viability than standard *S. cerevisiae*, while Capece et al. [78] noted no significant volatile profile changes yet observed higher antioxidant activity and polyphenol content compared to beer fermented with *Sacch. cerevisiae*. Estela-Escalante et al. [47] pioneered preliminary research into *S. bacillaris* for producing craft beer in the brewing industry. According to Canonico et al. [38], wild non-*Saccharomyces* yeasts offer emerging probiotic potential for developing a premium craft beer with minimal ethanol and pronounced aromatic characteristics.

Based on our findings, *T. delbrueckii* MI120 was the most suitable strain for brewing applications, characterized by its superior fermentation performance and sensory profile. At the same time, *S. bacillaris* MI125 demonstrated a strong probiotic potential, mainly due to its antimicrobial and health-supporting characteristics.

## 5. Conclusions

This study highlights the potential of *Torulaspora delbrueckii*, *Starmerella bacillaris*, and *Saccharomyces cerevisiae* strains as dual-purpose strains with both probiotic and brewing functionalities. Their resilience to gastrointestinal stress, non-hemolytic behavior, and antibiotic resistance support their suitability for safe probiotic applications. Among the yeasts, *S. bacillaris* demonstrated significant antibacterial activity against foodborne pathogens, further reinforcing its functional relevance. All the yeast strains showed a strong potential for brewing applications based on their physiological and metabolic profiles. The pilot-scale brewing trials identified *T. delbrueckii* as a promising probiotic starter due to its superior sensory profile, elevated polyphenol content, and significant antioxidant activity. These findings open new avenues for exploring these yeast strains as probiotic co-starters in the tailored development of functional beers, aligning with the concept of “the taste from somewhere, the taste of something”.

## Figures and Tables

**Table 1:** In vitro testing of the antibacterial activity of yeast strains against pathogenic bacteria, measured by the diameter of the inhibition zone (mm).

Pathogenic Bacteria	*T. delbrueckii* MI120	*S. bacillaris* MI125	*Sacch. cerevisiae* BB06
*Bacillus cereus* ATCC 11778	10.83 ± 1.04 ^a^	25.83 ± 0.85 ^c^	14.83 ± 0.62 ^b^
*Listeria monocytogenes* ATCC 13932	0.00 ± 0.00 ^a^	25.00 ± 1.63 ^c^	14.67 ± 0.47 ^b^
*Staphylococcus aureus* ATCC 33592	0.00 ± 0.00 ^a^	18.33 ± 0.47 ^c^	16.17 ± 0.47 ^b^
*Pseudomonas aeruginosa* ATCC 15442	0.00 ± 0.00 ^a^	25.33 ± 0.47 ^c^	18.67 ± 0.94 ^b^
*Salmonella enterica* subsp. *enterica* serovar Typhimurium ATCC 14028	0.00 ± 0.00 ^a^	26.67 ± 0.94 ^c^	16.67 ± 1.25 ^b^
*Salmonella enterica* subsp. *enterica* serovar Enteritidis ATCC 13076	0.00 ± 0.00 ^a^	24.33 ± 0.47 ^c^	18.50 ± 1.47 ^b^
*Escherichia coli* ATCC 8739	0.00 ± 0.00 ^a^	24.33 ± 1.25 ^c^	15.50 ± 1.08 ^b^
*Serratia marcescens* ATCC 14756	7.83 ± 0.76 ^a^	22.00 ± 1.41 ^c^	16.83 ± 1.03 ^b^

Results are reported as means ± standard deviations (SDs) based on three experiments, with significant variations (*p* < 0.05) among strains quantified by the Tukey B test from one-way analysis of variance (ANOVA) and marked by distinct letters.

**Table 2:** Evaluation of safety characteristics of yeast strains.

Yeast Strains	Antibiotics Susceptibility							Hemolytic Activity
	FLU-25	ITR-10	KCA-10	MCL-10	AMP-10	C-30	E-15	
*T. delbrueckii* MI120	S	R	S	S	R	R	R	γ
*S. bacillaris* MI125	R	R	R	R	R	R	R	γ
*Sacch. cerevisiae* BB06	R	R	R	R	R	R	R	γ

Legend: R—resistant; S—sensitive; FLU-25—Fluconazole; ITR-10—Itraconazole; KCA-10—Ketoconazole; MCL-10—Miconazole; AMP-10—Ampicillin; C-30—Chloramphenicol; E-15—Erythromycin; γ—Gamma hemolysis.

**Table 3:** In vitro screening of yeast strains for their functional abilities relevant to the brewing process.

Parameters		Yeast Strains			
		*T. delbrueckii* MI 120	*S. bacillaris* MI125	*Sacch. cerevisiae* BB06	*Sacch. cerevisiae* US-05
Carbon assimilation ^a^	2% Glucose	+	+	+	+
	2% Fructose	+	+	+	+
	2% Maltose	+	−	+	+
	2% Sucrose	+	+	+	+
Osmotic stress ^a^	30% Glucose	+	+	+	+
10% Malt extract tolerance with gas production ^b^		-	−	+	+
Ethanol tolerance ^a^	0%	+	+	+	+
	2.5%	+	+	+	+
	5%	+	+	+	+
	7.5%	+	−	+	+
Low pH tolerance ^c^		+++	+++	+++	+++
Acetic acid production ^d^		+	+	+	+
H_2_S production ^e^		brown	brown	brown	brown
Killer phenotype ^f^	Sensitive	−	−	−	+
	Killer	−	−	−	−

Legend: ^a^ Growth observed after 1 d of incubation: −, no growth; + biomass developed; ^b^ Gas production: no accumulation (−); gas accumulation (+); ^c^ Low pH tolerance: no growth (−), OD_600_ = 0.1; weak growth (+), 0.5 < OD_600_ < 1.0; strong growth (+++), OD_600_ > 1.0; ^d^ Halo: none (−); halo detected (+); ^e^ Biomass color: white, light tan, tan, brown, dark brown, and black; ^f^ killer phenotype: neutral (−); killer or sensitive phenotype (+).

**Table 4:** Enzymatic profile of yeast strains.

Enzyme	Substrate	*T. delbrueckii* MI 120	*S. bacillaris* MI125	*Sacch. cerevisiae* BB06	*Sacch. cerevisiae* US-05
Alkaline phosphatase	2-Naphthyl phosphate	1	1	1	
Esterase (C4)	2-Naphthyl butyrate	4	3	2	3
Lipase esterase (C8)	2-Naphthyl caprylate	3	2	2	2
Leucine arylamidase	L-Leucyl-2-naphthylamide	4	3	3	
Valine arylamidase	L-Valyl-2-naphthylamide	3	2	2	2
Cystine arylamidase	L-Cisteyl-2-naphthylamide	2	1	2	2
Acid phosphatase	2-Naphthyl phosphate	2	2	2	2
Naphthol-AS-BI-phosphohydrolase	Naphthol-AS-BI-phosphate	2	2	2	1
α-Galactosidase	6-Br-2-naphthyl-DD-galactopyranoside	2			1
β-Galactosidase	2-Naphthyl-ßD-galactopyranoside				1
β-Glucuronidase	Naphthol-AS-BI-ßD-glucuronide	1		1	1
α-Glucosidase	2-Naphthyl-DD-glucopyranoside				3
β-Glucosidase	6-Br-2-naphthyl-ßD-glucopyranoside		2		

Legend: color intensity from 0 (negative) to 5 (maximum intensity).

**Table 5:** Physico-chemical and technological analysis of beer samples.

Beer Samples	Ep, %P	Alcohol, % *v*/*v*	Bitterness, BU	Colour, EBC	Total Acidity, mL NaOH 1 N at 100 mL Beer	RDF, %
Beer fermented with *T. delbrueckii* MI120	11.08 ± 0.08 ^a^	4.21 ± 0.02 ^b^	28.00 ± 2.27 ^c^	9.00 ± 0.51 ^a^	1.60 ± 0.01 ^a^	59.17 ± 0.16 ^d^
Beer fermented with *S. bacillaris* MI125	10.90 ± 0.05 ^a^	3.36 ± 0.01 ^a^	28.00 ± 1.67 ^c^	9.19 ± 0.04 ^a^	2.23 ± 0.01 ^b^	48.27 ± 0.05 ^a^
Beer fermented with *Sacch. cerevisiae* BB06	10.97 ± 0.05 ^a^	3.50 ± 0.04 ^a^	26.00 ± 1.14 ^a^	9.78 ± 0.34 ^a^	2.00 ± 0.01 ^b^	49.76 ± 0.24 ^b^
Beer fermented with *Sacch. cerevisiae* US-05	11.37 ± 0.17 ^a^	4.26 ± 0.14 ^b^	27.00 ± 1.68 ^b^	9.69 ± 0.27 ^a^	1.80 ± 0.01 ^a^	58.38 ± 0.99 ^c^

Results are reported as means ± standard deviations (SDs) based on three experiments, with significant variations (*p* < 0.05) among strains quantified by the Tukey B test from one-way analysis of variance (ANOVA) and marked by distinct letters.

**Table 6:** Spectrum of sugars for wort and beer samples.

Beer Samples	Spectrum of Sugars, g/100 mL
Brewing wort used for the production of all beer samples	Glucose: 0.67 Sucrose: 0.54 Maltose: 2.81 Maltotriose: 0.89 Fructose: 0.06 Total sugars: 4.97
Beer fermented with *T. delbrueckii* MI120	Maltotriose: 0.043 Maltose: 0.019 Total sugars: 0.062
Beer fermented with *S. bacillaris* MI125	Maltose: 0.046 Fructose: 0.012 Total sugars: 0.058
Beer fermented with *Sacch. cerevisiae* BB06	Fructose: 0.010 Maltose: 0.021 Total sugars: 0.031
Beer fermented with *Sacch. cerevisiae* US-05	Fructose: 0.010 Total sugars: 0.010

**Table 7:** Total phenolic content and antioxidant activity of beer samples.

Beer Samples	TPC μg GAE·mL^−1^	Antioxidant Activity % RSA
Beer fermented with *T. delbrueckii* MI120	96.02 ± 4.57 ^b^	90.43 + 0.21 ^b^
Beer fermented with *S. bacillaris* MI125	76.59 ± 3.15 ^a^	90.53 + 0.37 ^b^
Beer fermented with *Sacch. cerevisiae* BB06	74.97 ± 1.69 ^a^	89.27 + 0.52 ^a^
Beer fermented with *Sacch. cerevisiae* US-05	93.65 ± 2.55 ^b^	91.05 + 0.31 ^b^

Legend: Results are reported as means ± standard deviations (SDs) based on three experiments, with significant variations (*p* < 0.05) among strains quantified by the Tukey B test from one-way analysis of variance (ANOVA) and marked by distinct letters.

## Data Availability

The original contributions presented in the study are included in the article, further inquiries can be directed to the corresponding author.
